# Divergence in intra-tumor variant frequencies in sporadic vestibular schwannomas as a potential indication for mosaic *NF2*-related schwannomatosis?

**DOI:** 10.1007/s10048-026-00903-5

**Published:** 2026-04-27

**Authors:** Lan Kluwe, Tabea Hartung, Steffen Rosahl, Said Farschtschi

**Affiliations:** 1 Department of Neurology, University Medical Center Hamburg-Eppendorf, Martinistr. 52, 20251 Hamburg, Germany; 2 Department of Neurosurgery, Campus Health and Medical University, Erfurt, Germany

**Keywords:** Mosaicism, Vestibular schwannoma, *NF2*, Variant allele frequency, Schwannomatosis

## Abstract

Vestibular schwannomas (VSs) are benign tumors of the eighth cranial nerve and the hallmark of *NF2*-related schwannomatosis. However, the majority of these tumors occur sporadically, arising from two inactivating alterations of the *NF2* gene. In this study, we re-evaluated a total of 29 VSs, each harboring two intragenic pathogenic *NF2* variants. In the six NF2-associated VSs, the intra-tumor variant frequencies differed substantially. Three of these cases were mosaic, as the pathogenic *NF2* variants were absent in the corresponding blood. By contrast, in the majority (20/23, 87%) of sporadic VSs, the allele frequencies of the two pathogenic *NF2* variants were nearly identical. Nevertheless, in two sporadic VSs, the intra-tumor variant frequencies were clearly divergent, suggesting the presence of cells with only one variant—the one with the higher allele frequency. We hypothesize that divergence in intra-tumor variant frequencies may provide a potential genetic indication for mosaic *NF2*-related schwannomatosis, even in cases that appear sporadic.

## Introduction

Vestibular schwannomas (VSs) are benign tumors of the eighth cranial nerve (vestibular nerve) that can impair hearing [1]. Approximately 5% of these tumors develop in the context of *NF2*-related schwannomatosis (hereafter referred to as NF2), a genetic disorder caused by heterozygous inactivation of the *NF2* tumor suppressor gene on chromosome 22. In the classical (non-mosaic) form of NF2, VSs typically present bilaterally and are often accompanied by other cerebral and spinal schwannomas, meningiomas and ependymomas [2, 3]. More than half of NF2 cases arise *de novo*, with mosaicism occurring in up to 60% [4, 5].

The majority of VSs occur sporadically and unilaterally in patients who do not meet the diagnostic criteria for NF2. However, some apparent sporadic VSs are in fact undiagnosed mosaic NF2, particularly in younger patients [4, 6].

In our recent sequencing study of 41 sporadic VSs [7], nine tumors harbored two distinct intragenic pathogenic *NF2* variants. In all nine, the intra-tumor allele frequencies of the two variants were nearly identical. Similar observations were made in the majority of cases from another study that sequencied VSs. However, three sporadic VSs in that study showed divergent intra-tumor variant frequencies. These findings led us to the hypothesis of possible mosaic NF2 among sporadic VSs.

### Data source

We re-evaluated data from three published studies [7–9], focusing on the frequencies of pathogenic variants. Only cases with two distinct intragenic pathogenic *NF2* variants were included: six associated with NF2 and 23 sporadic (Table 1). Variants of uncertain significance and allele-loss were excluded, as well as cases with more than two alterations which may present multifocal tumors. For all 9 VSs from our own study [7, 8] and 9 out of the 14 VSs from the other study [9], blood was sequenced. Copy number variations were assessed using multiplex ligation-dependent probe amplification.

**Table 1:** Allele-frequencies of pathogenic variants in sporadic and NF2-associated vestibular schwannomas

Patient ID	Age at onset (year)	Copy number variation	Type of variant^*^	Variant Allele Frequency (%)	Variant in blood	Total reads
Sporadic vestibular schwannoma [7, 8]						
2	35	no	Del 1 bp	30	no	1400
			SNV	29	no	300
3	59	no	Del 1 bp	26	no	180
			SNV	28	no	250
11	51	no	Del 1 bp	35	no	160
			SNV	37	no	280
28	56	no	Del 1 bp	24	no	800
			SNV	25	no	700
30	63	no	Del 1 bp	35	no	2800
			SNV	34	no	1700
33	62	no	Del 32 bp	11	no	1700
			Del 27 bp	11	no	1100
34	41	no	Del 1 bp	15	no	1900
			SNV	16	no	1900
39	27	no	Del 1 bp	24	no	2000
			SNV	21	no	2000
40	44	no	SNV	30	no	800
			SNV	28	no	3500
Sporadic vestibular schwannomas [9] supplemental dataset						
B02	23	no	SNV	34	no	Not available
			Del 2 bp	35	no	
B04	29	no	SNV	30	no	
			SNV	36	no	
B18	27	no	Del 4 bp	28	no	
			SNV	30	no	
B21	25	no	Ins 18 bp	**24**	no	
			Del 31 bp	**6**	no	
B26	27	no	SNV	29	not studied	
			Del 17 bp	31	not studied	
B27	22	no	SNV	27	not studied	
			Del 1 bp	22	not studied	
B29	27	no	Ins 17 bp	**28**	**no**	
			Del 1 bp	**42**	**no**	
B38	25	no	SNV	18	not studied	
			Del 1 bp	18	not studied	
C03	67	no	Del 2 bp	**37**	not studied	
			Del 17 bp	**14**	not studied	
C04	56	no	SNV	32	not studied	
			Del 1 bp	28	not studied	
C06	42	no	Del 1 bp	24	not studied	
			SNV	22	not studied	
C21	59	no	Del 1 bp	9	not studied	
			Del 8 bp	11	not studied	
C29	42	no	SNV	37	not studied	
			Del 45 bp	40	not studied	
C40	54	no	Del 1 bp	8	not studied	
			Del 1 bp	7	not studied	
NF2-associated vestibular schwannomas [9] supplemental dataset						
A04	17	no	Ins 1 bp	55	yes	Not available
			SNV	31	no	
A05	46	no	SNV	4	no	
			SNV	41	no	
A09	27	no	SNV	14	no	
			Del 33 bp	10	no	
A11	39	not studied	SNV	13	no	
			Del 3 bp	21	no	
A13	21	not studied	SNV	62	not studied	
			Del 1 bp	17	not studied	
A16	23	no	SNV	15	not studied	
			Del 4 bp	50	not studied	

^*^ Natures of the variants at sequences are given to consider the amplification efficiency of the variant-alleles. All variants are pathogenic. SNVs are single base variants causing stop-gain or alter the annotated splicing sequences. Deletion and Insertion are given with size in bp

## Results

In all six NF2-associated VSs, intra-tumor variant frequencies were clearly divergent. Blood samples were available for four patients; in three of these, the pathogenic *NF2* variants found in the tumor were absent in the corresponding blood, indicating mosaic NF2.

Among the 23 sporadic VSs, intra-tumor variant frequencies were nearly identical or very similar in 20 cases. In the remaining three cases (Table 1: B21, B29, C03), the frequencies diverged substantially. In case B29, the lower frequency (28%) of one variant may be explained by a 17-bp insertion likely reducing amplification efficiency. However, in cases B21 and C03, the divergence are unlikely attributed to differential amplification efficiency. An alternative interpretation is that they are mosaic NF2.

### Hypothesis

**Fig. 1:**
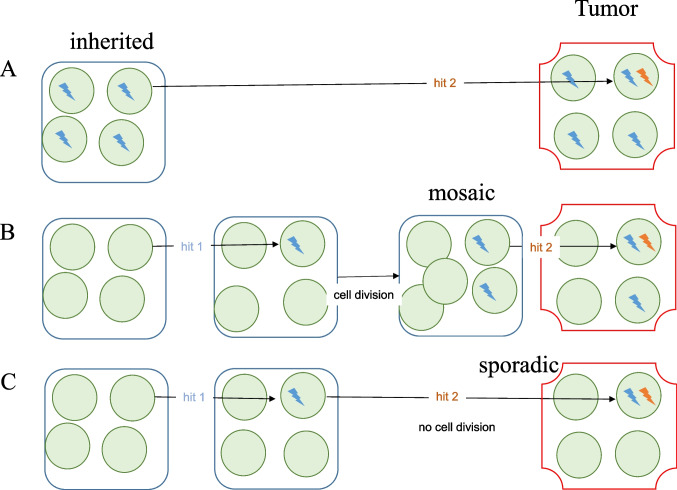
Two inactivating hits on a tumor suppressor gene leading to tumor development. **A** Inherited case where all cells carry the first hit. **B** Mosaic case with cell division between the first and second hits. **C** Truly sporadic tumor with no cell division between the first and second hits

According to the two-hit hypothesis for tumor suppressor genes [10], biallelic inactivation of *NF2* is the genetic driver of all VSs. In an inherited NF2 case, the first hit is present in all cells (Fig. 1A). A VS specimen from such a patient may contain two cell populations: (1) tumor cells harboring both hits, and (2) non-tumor cells carrying only the first hit (Fig. 1A).

In a mosaic NF2 case, the first hit occurs post-zygotically during early embryonic development, resulting in two cell populations in the patient—with and without the first hit (Fig. 1B). Consequently, a VS specimen from a mosaic NF2 patient may contain three cell populations: (1) tumor cells with two hits, (2) non-tumor cells with only the first hit, and (3) non-tumor cells with no hit (Fig. 1B). Due to the presence of non-tumor cells with the first hit, a VS from a mosaic NF2 patient is expected to show divergent intra-tumor variant frequencies.

In a truly sporadic VS, the first and second hits occur in the same cell simultaneously or without intervening cell division (Fig. 1C). This scenario is plausible when the first hit occurs later in life, after nerve development is complete and Schwann cells have ceased proliferation. A specimen from a truly sporadic VS would therefore contain only two cell populations: (1) tumor cells with both hits, and (2) non-tumor cells with no hit (Fig. 1C). As a result, the intra-tumor variant frequencies are nearly identical which reflects the proportion of tumor cells in the specimen. This pattern aligns with our observations in the majority (20/23) of sporadic VSs (Table 1).

However, it is also possible that the first hit occurs alone in a cell, which then divides several times before the second hit occurs later in one of the daughter cells (Fig. 1B). Theoretically, this scenario corresponds to a mosaic case, even if the cell population carrying only the first hit is small. The resulting VS would display divergent allele frequencies (Fig. 1B).

According to the above hypothesis, divergence in intra-tumor variant frequencies may serve as a potential genetic indicator of mosaic NF2, also for cases that appear sporadic.

#### Considerations

Several factors may also influence variant frequencies. First, larger deletions or insertions, as well as variants located near amplicon ends, may affect amplification efficiency. Second, poor DNA quality or quantity and insufficient sequencing coverage can lead to variability that mimics divergent frequencies [11]. Third, the presence of multifocal tumors or contamination with unrelated samples may produce divergent frequencies that mimic a mosaic pattern. To mitigate these issues, we recommend repeat sequencing using newly prepared samples, higher coverage, and multiple distinct amplicons for each variant. High-quality DNA from native specimens is preferred [8]. Analysis of additional tissues, such as buccal cells or hair follicles, may also be considered.

#### Clinical and research implications

Our hypothesis is primarily theoretical and supported by limited evidence at this stage. Nevertheless, we believe that it is worth consideration and further investigation. Previously generated raw sequencing data with adequate coverage (> 500 ×) could be re-evaluated with attention to variant frequencies. Accumulating such data for large number of sporadic VSs will shed light on this issue. This hypothesis may extend beyond NF2 to other tumor suppressor syndromes, such as neurofibromatosis type 1.

Sequencing of sporadic VSs may be recommended for young patients, those with rapidly growing or large tumors, or those presenting with any NF2-associated features (e.g., cataracts, skin tumors). If a genetic indication for possible mosaic NF2 arises, additional clinical evaluations—such as high-resolution brain and spine MRI or ophthalmological examination—should be considered. Follow-up may also be beneficial as symptoms may develop later in mosaic cases [6].

## Data Availability

No datasets were generated or analysed during the current study.
